# Integrated Analysis of Proteomics and Metabolomics Uncovered the Anti-Inflammatory Mechanisms of Baicalin in CIA Rat FLS

**DOI:** 10.3390/cimb48010111

**Published:** 2026-01-20

**Authors:** Li Wang, Si Yao, Jing Wang, Yuxin Yang, Tiansong Wang, Maiyan Hai, Wei Zhang, Na Wang, Qiaofeng Wan

**Affiliations:** 1 Department of Rheumatology, The First Clinical Medical College, Ningxia Medical University, Yinchuan 750003, China; niuxiaoniu@163.com; 2 Department of Pathogenic Biology and Immunology, College of Basic Medical Science, Ningxia Medical University, Yinchuan 750004, China; 18711717258@163.com (S.Y.); 17835684863@163.com (J.W.); yangyuxin19971002@126.com (Y.Y.); wencheng1998211@163.com (T.W.); hmyhmy0821@126.com (M.H.); zhangwei@nxmu.edu.cn (W.Z.); 20150130@nxmu.edu.cn (N.W.)

**Keywords:** baicalin, rheumatoid arthritis (RA), fibroblast-like synoviocytes (FLS), proteomics, metabolomics

## Abstract

Rheumatoid arthritis (RA) is a chronic autoimmune disorder characterized by persistent synovitis, in which fibroblast-like synoviocytes (FLSs) serve as the primary effector cells that drive the destruction of joints. Baicalin has previously demonstrated efficacy in significantly ameliorating joint symptoms in rats with CIA. As such, this study aims to investigate its underlying molecular mechanisms and impact on the FLSs of rats with CIA through an integrated proteomics and transcriptomics analysis. A Kyoto Encyclopedia of Genes and Genomes (KEGG) pathway analysis was conducted based on two datasets; it revealed that the retrograde endocannabinoid signaling pathway—associated with susceptibility to RA—is the only one involved in both the signaling and metabolic processes modulated by baicalin. Nineteen differentially expressed proteins (DEPs) downregulated by baicalin comprise seventeen subunits of NADH dehydrogenase and two receptors, glutamate receptor 2 (GRIA2) and γ-aminobutyric acid receptor subunit alpha-5 (GABRA5). Three differential metabolites (DMs) were also affected by baicalin: γ-aminobutyric acid (GABA) and phosphatidylcholine (PC) were upregulated and phosphatidylethanolamine (PE) was downregulated. Our findings suggest that the baicalin-mediated alleviation of joint synovitis is closely related to the upregulation of GABA and PC; downregulation of GRIA2, GABRA5, and PE; and preservation of mitochondrial homeostasis within the retrograde endocannabinoid signaling pathway in FLSs.

## 1. Introduction

Rheumatoid arthritis (RA) is a chronic, systemic autoimmune disease marked by persistent inflammatory synovitis and progressive joint destruction [1]. It affects approximately 0.5–1% of the global adult population and can reduce life expectancy by up to 10 years or more compared to the general population [2,3]. Fibroblast-like synoviocytes (FLSs) are the primary effector cells that destroy cartilage and bone in RA-affected joints; this destruction occurs through crosstalk with inflammatory cells and direct damage to tissue pathways [4,5]. Recent evidence suggests that FLSs in RA synovial tissue exhibit significant differences in metabolism compared to those in normal synovial tissue [6]. Much progress has been made in developing disease-modifying antirheumatic drugs (DMARDs). They can be categorized into three major classes: (1) conventional synthetic DMARDs, such as methotrexate (MTX), leflunomide, and sulfasalazine; (2) targeted synthetic DMARDs, including Janus kinase inhibitors such as tofacitinib, baricitinib, and upadacitinib; and (3) biological DMARDs, such as tumor necrosis factor inhibitors, including adalimumab, certolizumab pegol, etanercept, golimumab, and infliximab [7,8]. Although these agents effectively alleviate symptoms and slow disease progression, their widespread use is limited by high costs and potential adverse effects [9,10]. Therefore, there is an urgent need to develop novel therapeutic agents that are both cost-effective and associated with minimal toxicity.

Baicalin is a flavonoid isolated from the root of Scutellaria baicalensis; it exhibits multiple pharmacological properties and is associated with low toxicity [11]. Following evidence of its efficacy in significantly ameliorating joint symptoms in rats with CIA, this study aims to investigate its underlying molecular mechanisms and effect on the FLSs of rats with CIA through an integrated proteomics and transcriptomics analysis.

## 2. Materials and Methods

### 2.1. Animals and CIA Model Preparation

In this study, 24 male Sprague-Dawley (SD) rats (7 weeks old; weight 150 ± 10 g) were purchased from the Experimental Animal Center of Ningxia Medical University (certificate no. SCXK, 2020-0001), and the use of these animals was approved by the Ethics Committee of Ningxia Medical University (approval no. 2023-N0023; approval date: 8 March 2023). The rats were raised in a standard pathogen-free environment with a temperature of 22 ± 2 °C, humidity of 55 ± 5%, 12 h light/dark cycles and free access to food and water. For the preparation of the rats used in the CIA model, 0.1 mL of bovine collagen II (Chondrex, Inc., Washington, DC, USA) emulsified in Freund’s complete adjuvant (C55-R10291; Shanghai Canspec Scientific Instruments Co., Ltd., Shanghai, China) was administered twice at 7-day intervals via subcutaneous injection into the base of the tail and hind footpads. Then, the degree of hind feet erythema and swelling was assessed with 5-grade arthritis scoring [11]. At day 21 after primary immunization, rats with an arthritis score of >2 were selected for the CIA model.

### 2.2. Baicalin Treatment and Therapeutic Efficacy Assessment

At day 21 after the initial immunization, the rats with CIA were randomly divided into the model group, baicalin-treated group and dexamethasone (DEX)-treated group. Rats in the baicalin group were administered the flavonoid (60 mg/kg/day; baicalin purity ≥99%; Kehua AI Innovative Drug Research and Development Co., Ltd., Wuhan, China) and those in the DEX group received DEX (0.5 mg/kg/day; H41020035; Sinopharm Ronshyn Pharmaceutical Co., Ltd., Jiaozuo, China), both via intraperitoneal injection once a day. In the normal and model groups, rats were given an equivalent volume of saline administered via an intraperitoneal injection. Mouse weight, joint swelling, and arthritis index score were measured. After 21 days of treatment, the rats underwent an 8 h fasting period (with free access to water) and were then anesthetized with ether to collect blood before being sacrificed via cervical dislocation. Then, synovial tissue from the hind knee joints of the rats with CIA was dissected to isolate and culture FLSs.

### 2.3. Primary Culture of Fibroblast-like Synoviocytes [12] and Baicalin Treatment

Adipose tissue and other connective tissues were removed from synovial tissue and then rinsed thoroughly three times with D-Hanks’ solution and minced into fragments measuring 1–2 mm^3^. The minced tissue was washed with DMEM supplemented with 20% fetal bovine serum until the supernatant became clear. The tissue fragments were evenly distributed at the bottom of a culture flask, which was then carefully inverted and incubated at 37 °C in a humidified atmosphere containing 5% CO_2_ for 2 h to facilitate tissue adherence. Thereafter, prewarmed DMEM containing 20% fetal bovine serum was gently added, ensuring that the medium just covered the tissue explants, and the culture medium was replaced every 2 to 3 days. Once a substantial number of fibroblast-like cells migrated out from the tissue fragments, the remaining tissue blocks were removed to allow the remaining adherent cells to continue culturing. Fibroblast-like synoviocytes (FLSs) were subcultured when they reached a confluent state, and third-generation cells were used for subsequent in vitro experiments. FLSs were treated with baicalin at a working concentration of 50 µg/mL for 48 h at 37 °C, while untreated FLSs served as the control group.

### 2.4. Detection of Inflammatory Factors by Enzyme-Linked Immunosorbent Assay (ELISA)

Serum levels of IL-6, IL-10 and IL-17 were determined using ELISA kits (Hejun Biotechnology Co., Ltd., Wuhan, China) according to the manufacturer’s instructions.

### 2.5. Hematoxylin and Eosin (H&E) Staining of the Ankle Joints

The ankle joints of the hind limbs were fixed in 4% paraformaldehyde solution for 24 h and were then subject to formalin EDTA (Ethylene Diamine Tetraacetic Acid) decalcifying solution (BL518A; Biosharp, Beijing, China) for 2 months, with the solution changed once a week. After the joint tissues were embedded in paraffin and cut into 5 μm thick sections, HE staining was performed.

### 2.6. Identification of Differentially Expressed Proteins (DEPs) with Proteomics Analysis

Proteomics analysis was conducted by PTM Biolabs Inc. (Hangzhou, China). Cell samples were lysed via sonication in lysis buffer containing 8 mol/L urea (Sigma-Aldrich, St. Louis, MO, USA), utilizing a high-intensity ultrasonic processor (Scientz, Ningbo, China) and a protease inhibitor cocktail at a concentration of 0.01 g/mL (Merck Millipore, Darmstadt, Germany). Following centrifugation, the supernatant was collected after removing cellular debris. Protein concentration in the supernatant was determined using a BCA protein assay kit (Solarbio, Beijing, China). Prior to the digestion of trypsin, proteins were subjected to reduction with dithiothreitol, alkylation with iodoacetamide, and dilution with tetraethylammonium bromide. The resulting peptides were desalted using Strata X C18 SPE columns (Phenomenex, Torrance, CA, USA) and labeled with a TMT labeling kit (Thermo Fisher Scientific, Waltham, MA, USA). They were separated into fractions using an Agilent 300 Extend C18 column as part of a high-performance liquid chromatography (HPLC) system. The fractionated peptides were then reconstituted in acetonitrile (Thermo Fisher Scientific, Waltham, MA, USA) and analyzed using a Q Exactive HF-X mass spectrometer (Thermo Fisher Scientific, Waltham, MA, USA). RAW data were processed using MaxQuant software (v.1.5.2.8, Max Planck Institute of Biochemistry, Martinsried, Germany) against the rattus norvegicus proteome database obtained from UniProt. Proteomics experiments were performed with three biological replicates.

### 2.7. Metabolomics Analysis

Cell samples were collected, and 1 mL of a solvent mixture comprising methanol, acetonitrile, and ddH2O (in a 2:2:1 volume ratio) was added to each sample. Subsequently, the samples were subjected to ultrasonication for 10 min in an ice bath, followed by centrifugation at 13,000 rpm for 15 min at 4 °C. The resulting supernatants were analyzed using the Thermo Fisher Scientific Vanquish system equipped with a Waters ACQUITY UPLC BEH Amide column (2.1 mm × 50 mm, 1.7 μm). The mass spectrometric parameters were set as follows: the sheath gas flow rate at 50 arbitrary units (Arb), the auxiliary gas flow rate at 15 Arb, capillary temperature at 320 °C, full MS resolution at 60,000, and MS/MS resolution at 15,000. The collision energy was configured to stepped normalized collision energy (SNCE) levels of 20/30/40, and the spray voltage was set to 3.8 kV in positive ion mode and −3.4 kV in negative ion mode. Integrated metabolomic and proteomic analyses were conducted, and DEPs and DMs were filtered using a threshold of >1.5-fold and *p* < 0.05. The filtered datasets were then imported into the Venny2.1 tool to visualize overlapping features across the datasets. Species-specific pathway analysis was performed using the KEGG pathway database (www.kegg.jp/kegg/pathway.html (accessed on 16 July 2025)), and metabolomics experiments were conducted with three biological replicates.

### 2.8. Western Blot Analysis

Proteins were extracted from each group of cells and quantified using a BCA protein assay kit (Beyotime, Shanghai, China). Equal amounts of protein samples were separated using 10% SDS-PAGE and subsequently transferred onto PVDF membranes. The membranes were blocked with 5% non-fat milk and then incubated overnight at 4 °C with the following primary antibodies: anti-NDUFV1 (cat. E-AB-11436; Elabscience, Wuhan, China), anti-GRIA2 (cat. db11905; Diagbio, Hangzhou, China), anti-GABRA5 (cat. R383011; Zenbio, Chengdu, China), anti-β-Actin (cat. AC026; ABclonal, Wuhan, China), and anti-GAPDH (cat.BOAR-00007-A; Biosharp, Beijing, China). Following three washes with TBST, the membranes were incubated with the corresponding secondary antibody (cat. SA00001-2; Proteintech, Wuhan, China) at 37 °C for 60 min. Protein bands were then detected using the New Super ECL Assay (cat. BL520B; Biosharp, Beijing, China).

### 2.9. Fluorescence Analysis

FLS cells were seeded in 24-well plates covered with cell slivers at a density of 2 × 104 cells/well, with three replicate wells per group, and cultured for 24 h. Following 48 h treatment with baicalin, the cell slivers were removed, and the cells were fixed with 4% paraformaldehyde for 15 min. Subsequently, they were permeabilized with 0.25% Triton X-100 for 10 min and blocked with 3% BSA at 37 °C for 30 min. Primary antibodies, anti-NDUFV1, anti-GRIA2 and anti-GABRA5, were then added and incubated at 37 °C for 1 h. A fluorescent secondary antibody (cat. SA00014-5; Proteintech, Wuhan, China) was applied dropwise, followed by incubation in the dark for 30 min. Finally, the nuclei were stained with DAPI, and the samples were mounted for observation and imaging using a fluorescence microscope.

### 2.10. Bioinformatics and Statistical Analysis

All identified proteins were annotated using GO and the KEGG database. Data were expressed as mean ± standard deviation (SD) using GraphPad Prism 6 (GraphPad Software, Inc., San Diego, CA, USA). Statistical significance between two groups was determined by Student’s *t*-test and among three or more groups by one-way analysis of variance (ANOVA) followed by post-hoc testing. *p*-value < 0.05 was statistically significant.

## 3. Results

### 3.1. Baicalin Relieved Joint Swelling, Improved Body Weight, Reduced Arthritis Scores, Reduced the Levels of Pro-Inflammatory Cytokines, and Reduced Inflammatory Infiltration of the Joint Synovium in CIA Rats

**Figure 1:**
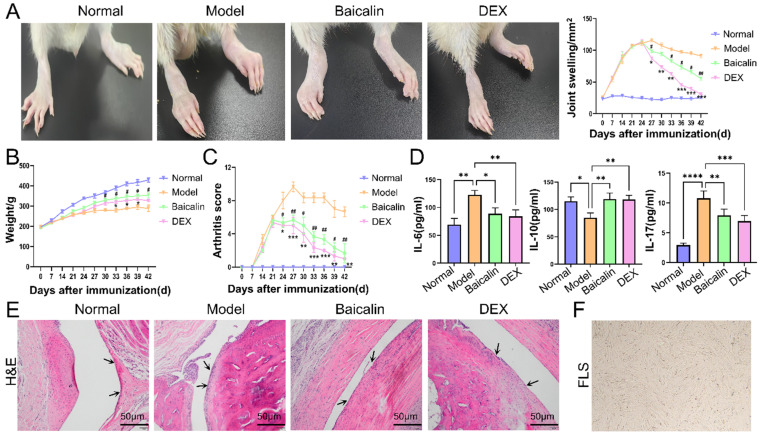
Assessment of the Therapeutic Efficacy of Baicalin in Joint Inflammation. (**A**) Representative picture of hind paws and paw swelling. (**B**) Baicalin improved the weight, which was measured every three days. (**C**) Baicalin reduced the arthritis score, which was measured every three days. (**D**) Baicalin reduced the levels of IL-6 and IL-17, while elevating the level of IL-10. (**E**) Baicalin alleviated inflammation in the synovial tissue by HE staining. The arrow indicates synovial tissue. (**F**) Primary cultured third-generation fibroblast-like synoviocytes (10×). Data are expressed as means ± SD (*n* = 6). * *p* < 0.05, ** *p* < 0.01,*** *p* < 0.001, **** *p* < 0.0001; ^#^ *p* < 0.05, ^##^ *p* < 0.01.

To evaluate the efficacy of baicalin in treating inflammation associated with RA, we employed the widely used CIA rat model characterized by elevated joint swelling and arthritis scores (Figure 1A). Compared to the model group, rats treated with baicalin exhibited greater weight improvement from the 30th day (*p* < 0.05) and had significantly reduced arthritis scores from the 24th day (*p* < 0.05), reduced levels of IL-6 (*p* < 0.05) and IL-17 (*p* < 0.01), and higher levels of IL-10 (*p* < 0.01) at the end (Figure 1B–D). To evaluate the effect of baicalin on inflammatory infiltration in joint synovial tissue, hind ankle joints were stained using H&E. In comparison to the model group, inflammatory infiltration in the synovial tissue was markedly reduced in the baicalin-treated group (Figure 1E). Furthermore, fibroblast-like synoviocytes (FLSs) were successfully isolated and cultured from the synovial tissue of knee joints in rats with CIA, with third-generation cells utilized for subsequent experiments (Figure 1F).

### 3.2. Analysis of DEPs with TMT-Based Quantitative Proteomics

**Figure 2:**
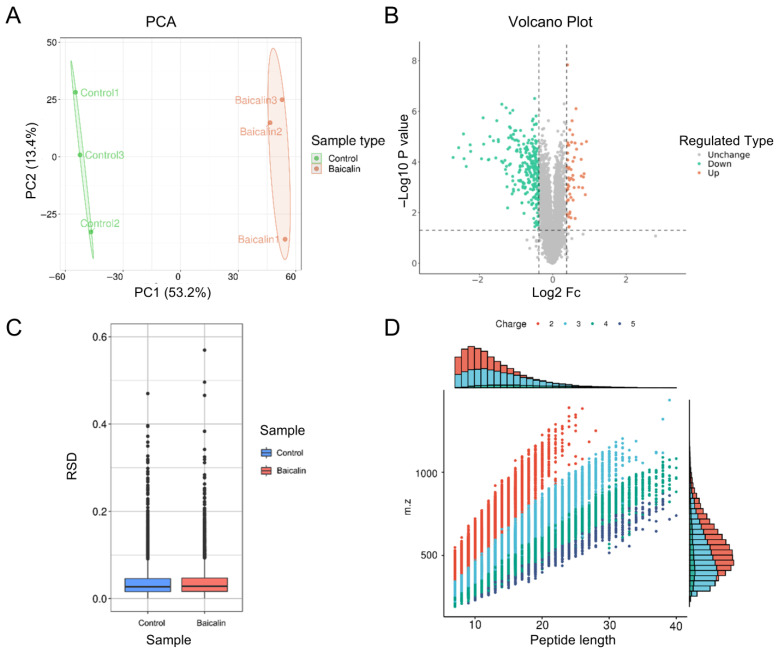
Analysis of differentially expressed proteins (DEPs) with TMT-based quantitative proteomics. (**A**) The degree of aggregation between the groups based on PCA. (**B**) Volcano plot. Red bubbles represent upregulated proteins and blue bubbles represent downregulated proteins. (**C**) Relative Standard Deviation between the groups. (**D**) The length distribution of the peptides.

To investigate baicalin-regulated proteins and their molecular mechanisms in rheumatoid arthritis (RA), TMT-based proteomics was employed to identify differences in DEPs between the control and baicalin-treated groups. The results from principal component analysis (PCA) demonstrated that the proteomes in both groups were distinguishable (Figure 2A), indicating that baicalin treatment significantly altered proteomic profiles. Moreover, 52 upregulated and 264 downregulated proteins were identified in the baicalin group compared to the control, using a threshold of fold change >1.5 and *p*-value < 0.05 (Figure 2B). The degree of variation between the two groups reflects the stability and reliability of the experimental results (Figure 2C). Additionally, most peptide segments were found within the range of 7 to 20 amino acids, meeting the quality control standards (Figure 2D).

### 3.3. Functional Classification of the Baicalin-Mediated DEPs

**Figure 3:**
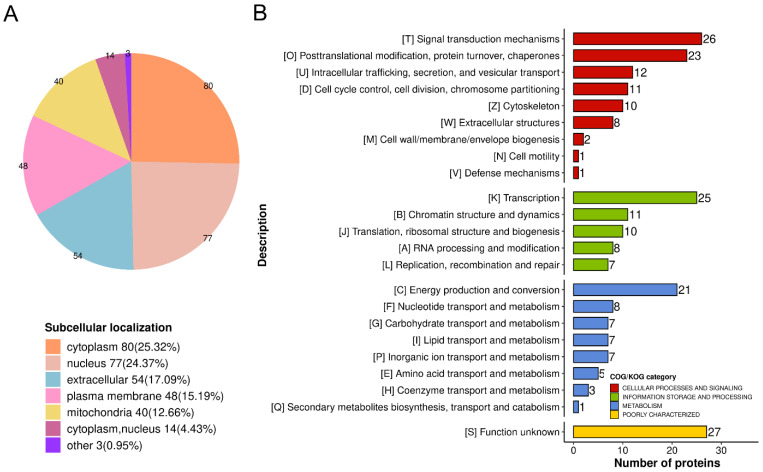
Classification of 316 differentially expressed proteins (DEPs) in the Baicalin groups and Control groups. (**A**) Subcellular localization classification. (**B**) Clusters of orthologous groups (COG)/clusters of eukaryotic orthologous groups (KOG) of DEPs.

To analyze the functions of baicalin-related DEPs, functional classification was conducted based on their subcellular localization and cluster of orthologous groups (COG) annotation. The results indicated that the DEPs were localized predominantly in the cytoplasm (25.32%), nucleus (24.37%), extracellular space (17.09%), plasma membrane (15.19%), mitochondria (12.66%), cytoplasm–nucleus (4.43%), and other unspecified locations (0.95%) (Figure 3A). According to the COG classification, baicalin-related proteins were primarily involved in cellular processes and metabolism. Functional categories included signal transduction mechanisms, post-translational modifications, transcription, energy production and conversion, and information storage and processing (Figure 3B).

### 3.4. Functional Enrichment Analysis of the Baicalin-Related DEPs

**Figure 4:**
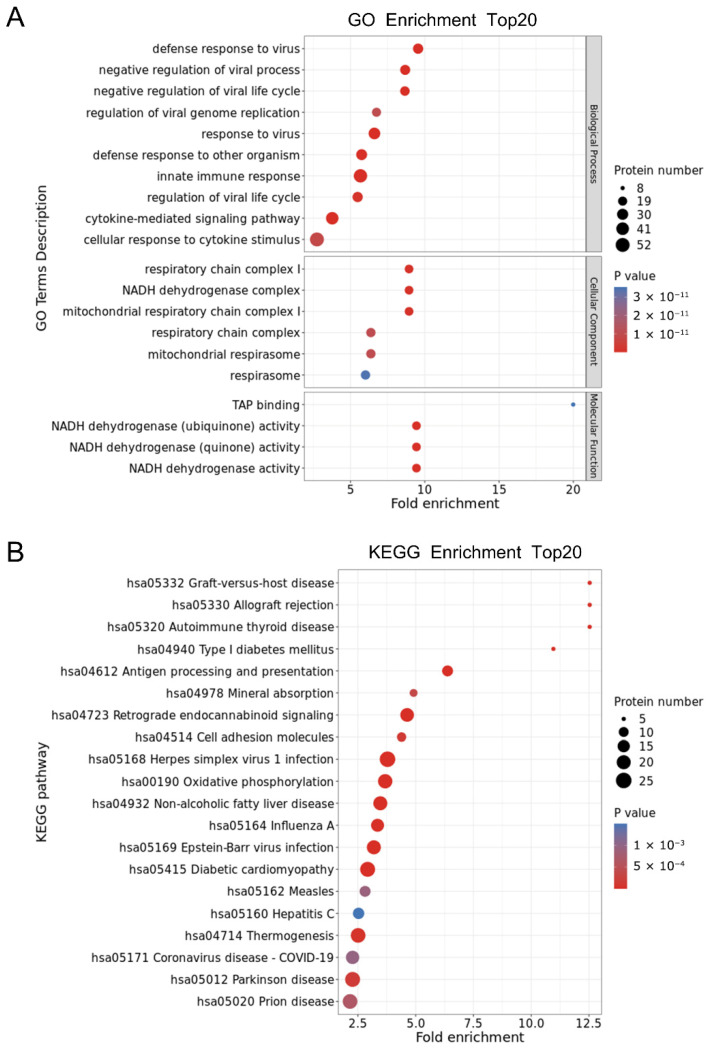
Functional enrichment and cluster analysis of differentially expressed proteins (DEPs) in the Baicalin and Control groups. (**A**) Gene Ontology (GO) enrichment analysis. The size of the dot represents the number of enriched genes and the color represents the significance level. (**B**) Kyoto Encyclopedia of Genes and Genomes (KEGG) pathway. The size of the dot represents the number of enriched genes and the color represents the significance level.

In total, 316 DEPs were identified in the control and baicalin groups and were subjected to Gene Ontology (GO) enrichment analysis (Figure 4A). The results illustrate the functional classification of the top 20 most significantly enriched categories. In terms of biological processes, the DEPs were predominantly enriched in innate immune response, viral response, and cytokine-mediated signaling pathways. With respect to cellular components, significant enrichment was observed in mitochondrial respiratory chain complex I, the NADH dehydrogenase complex, and respiratory chain complex I. The primary molecular functions exhibiting enrichment were NADH dehydrogenase (quinone) and NADH dehydrogenase (ubiquinone) activity. Furthermore, KEGG pathway enrichment analysis was conducted on the same set of 316 DEPs (Figure 4B), revealing a total of 53 enriched pathways; the figure highlights the top 20 signaling pathways with the most significant enrichment. Notably, the DEPs were primarily associated with pathways such as hsa05168: herpes simplex virus 1 infection; hsa04723: retrograde endocannabinoid signaling; and hsa00190: oxidative phosphorylation.

### 3.5. Effects of Baicalin on Differential Metabolites (DMS) in FLS

**Figure 5:**
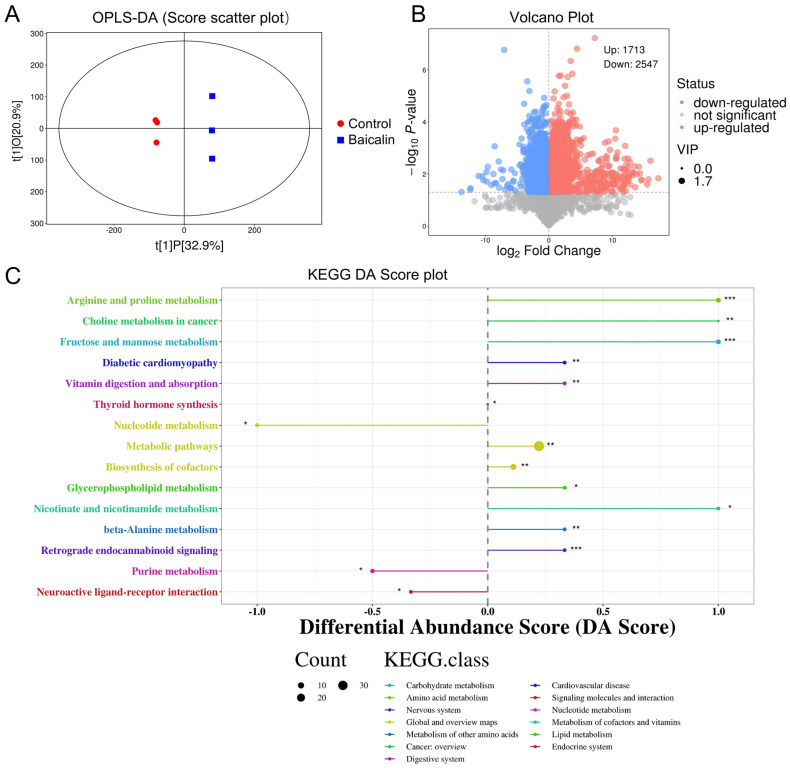
Effects of baicalin on differential metabolites (DMs) in FLS. (**A**) OPLS-DA score plot, showing separation between Control (red) and Baicalin (blue) groups. (**B**) The metabolite volcano plot. Red bubbles represent upregulated metabolites and blue bubbles represent downregulated metabolites. (**C**) KEGG differential abundance score, quantifying differences in the abundance of metabolites within specific KEGG pathways between the Baicalin and control groups. * *p* < 0.05, ** *p* < 0.01,*** *p* < 0.001.

To further explore the effects of baicalin on metabolic processes in FLS cells, differential metabolites (DMs) in the baicalin and control groups were systematically examined. Multifaceted analytical approaches, including OPLS-DA (Figure 5A) and volcano plot analysis (Figure 5B), were applied. A total of 22,895 metabolites were identified, among which 4260 were classified as DMs, comprising 1713 significantly upregulated and 2547 significantly downregulated DMs. Subsequent metabolic pathway analysis based on KEGG differential abundance scores revealed that the most significantly altered pathways were arginine and proline metabolism, fructose and mannose metabolism, and retrograde endocannabinoid signaling (Figure 5C).

### 3.6. Integrated Analysis of the DEPs and DMs and Regulated by Baicalin

**Figure 6:**
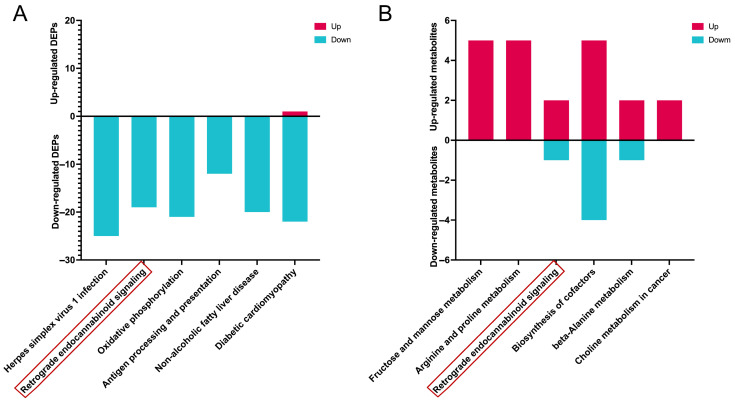
Analysis of differentially expressed proteins (DEPs) and differentially metabolites (DMs) between the Baicalin and Control groups. (**A**) Proteins KEGG enrichment analysis. Red columns represent upregulated proteins and blue columns represent downregulated proteins. (**B**) Metabolites KEGG enrichment analysis. Red columns represent upregulated metabolites and blue columns represent downregulated metabolites. The red box indicates the common signaling pathways regulated by baicalin.

To elucidate the DEPs and DMs regulated by baicalin, an integrated analysis was conducted using proteomic and metabolomic data from the control and baicalin groups, and these datasets were subjected to KEGG pathway enrichment analysis. Figure 6A presents the six main signaling pathways and corresponding number of DEPs regulated by baicalin, while Figure 6B illustrates the six primary metabolic pathways and associated DM counts. Among all identified pathways modulated by baicalin, the only one that appears in both the DEP signaling process and metabolic profiles is retrograde endocannabinoid signaling (Figure 6).

### 3.7. The DEPs and DMs Regulated by Baicalin in Retrograde Endocannabinoid Signaling

In retrograde endocannabinoid signaling, nineteen DEPs are downregulated by baicalin, as presented in Table 1. Among these proteins, seventeen subunits are components of NADH dehydrogenase, and two are receptors, specifically, Glutamate Receptor 2 and Gamma-Aminobutyric Acid Receptor Subunit Alpha-5 (GABRA5). Additionally, three DMs regulated by baicalin—two upregulated and one downregulated—are listed in Table 2.

### 3.8. Confirmation of the NDUFV1, GRIA2 and GABRA5

**Figure 7:**
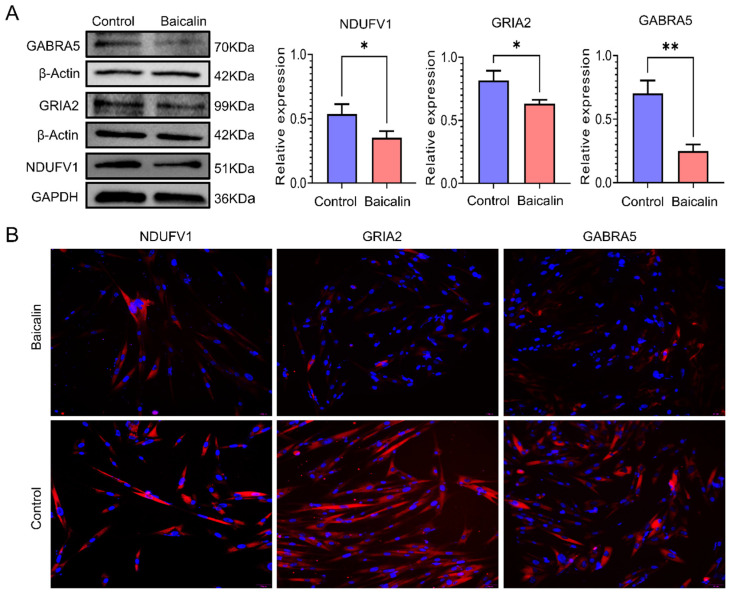
Confirmation of the NDUFV1, GRIA2 and GABRA5 by Western blot and fluorescence. (**A**) Representative Western blot images of NDUFV1, GRIA2 and GABRA5, and their statistical plots. (**B**) Fluorescence photos for NDUFV1, GRIA2 and GABRA5. All data were expressed as mean ± standard deviation (SD) (*n* = 3). * *p* < 0.05, ** *p* < 0.01.

NDUFV1, GRIA2, and GABRA5 were selected for further analysis. Western blot and fluorescence analyses demonstrated that baicalin down-regulated the expression levels of NDUFV1, GRIA2, and GABRA5, which is consistent with the results obtained from the integrated analysis (Figure 7A,B).

## 4. Discussion

Rheumatoid arthritis (RA) is an autoimmune disease characterized by chronic inflammation and the progressive destruction of joints and systemic organs [13]. The primary pathological features of RA include persistent synovial hyperplasia and the continuous secretion of inflammatory cytokines within affected joints [14], both of which serve as key therapeutic targets for the development of novel anti-RA agents [15]. Fibroblast-like synoviocytes (FLSs) are the predominant cell type in synovial tissue. Upon abnormal activation, they exhibit a highly invasive phenotype, mediating inflammatory responses and contributing to joint damage [16,17]. Furthermore, the inflammatory microenvironment is significantly influenced by the glycolytic activity of rheumatoid arthritis fibroblast-like synoviocytes (RA-FLSs) and alterations in the metabolism of key macromolecules such as amino acids, glucose, and lipids [18]. These metabolic changes are closely associated with aberrant FLS activation and synovial inflammation [19]. Mitochondria are central organelles responsible for cellular energy production and play a crucial role in metabolic regulation [20]. Disruptions in mitochondrial homeostasis can affect synovial cell proliferation and modulate inflammatory signaling pathways implicated in the onset and progression of RA [21]. Additionally, studies have shown that patients with RA exhibit a higher frequency of mitochondrial mutations in synoviocytes and synovial tissues compared to those with osteoarthritis [22,23]. Therefore, targeting mitochondrial homeostasis holds promise for treating RA.

We found that baicalin alleviates the inflammatory infiltration of the joint synovium in rats with CIA, and baicalin suppresses the secretion of pro-inflammatory cytokines in RA-FLS cells [11]. As such, this study aimed to treat FLSs in rats with CIA with baicalin and further investigate the potential mechanism underlying the baicalin-mediated alleviation of joint synovitis through an integrated proteomics and transcriptomics analysis.

In this investigation, 52 upregulated and 264 downregulated proteins were identified following baicalin treatment. These differentially expressed proteins (DEPs) underwent KEGG pathway enrichment analysis, revealing significant enrichment in pathways such as hsa04723 retrograde endocannabinoid signaling, hsa00190 oxidative phosphorylation, and hsa05168 herpes simplex virus 1 infection. A total of 4260 differential metabolites (DMs) were detected, including 1713 significantly upregulated and 2547 significantly downregulated metabolites. The most significantly altered metabolic pathways included arginine and proline metabolism, fructose and mannose metabolism, and retrograde endocannabinoid signaling.

Proteomic and metabolomic data from the control and baicalin-treated groups were analyzed using KEGG pathway enrichment. Among the signaling and metabolic pathways modulated by baicalin, retrograde endocannabinoid signaling, which is associated with the risk of RA [24], was identified as the only pathway commonly involved in both signal transduction and metabolic processes. Within this pathway, nineteen DEPs regulated by baicalin were identified, including seventeen subunits of NADH dehydrogenase—also known as mitochondrial complex I [25]—and two receptors. The seventeen subunits consisted of NADH dehydrogenase flavoprotein 1 (NDUFV1), NADH dehydrogenase iron–sulfur proteins (NDUFS1, 4, 5, 6, 7), NADH dehydrogenase 1 alpha subcomplex subunits (NDUFA3, 5, 7, 8, 9, 10, 12), NADH dehydrogenase 1 subunits C (NDUFC1, 2), and NADH-ubiquinone oxidoreductase chains (MT-ND2, 3). The two receptors identified were glutamate receptor 2 (GRIA2) and γ-aminobutyric acid receptor subunit alpha-5 (GABRA5). In terms of metabolic changes within the retrograde endocannabinoid pathway, three DMs were found to be regulated by baicalin: γ-aminobutyric acid (GABA) and phosphatidylcholine (PC) were upregulated and phosphatidylethanolamine (PE) was downregulated.

Among the 17 NADH dehydrogenase subunits regulated by baicalin, NDUFV1 (ubiquinone oxidoreductase core subunit V1), a critical component of mitochondrial complex I [26], exhibited the most significant modulation. The abnormal expression of NDUFV1 has been closely linked to the pathogenesis of RA [27]. Within mitochondria, the synthesis and insertion of iron–sulfur (Fe/S) clusters into apoproteins are facilitated by a set of 17 proteins collectively referred to as the ISC (iron–sulfur cluster) assembly machinery [28]. The depletion of NDUFS7 has been shown to reduce cell proliferation and increase apoptosis [29]. NDUFA subunits are accessory components of mitochondrial complex I and are potential immunotherapeutic targets for modulating the tumor microenvironment [30]. NDUFC subunits contribute to the structural stability and biogenesis of complex I; the knockdown of NDUFC1 has been demonstrated to inhibit cell proliferation, migration, and invasion in hepatocellular carcinoma [31]. Mitochondrial-encoded NADH dehydrogenase (MT-ND) mutations have been implicated in the pathogenesis of rheumatoid arthritis (RA), with alterations in the mitochondrial genome playing a pivotal role in disease progression [22,32]. This study demonstrates that baicalin possesses the potential to restore the structure and function of NADH dehydrogenase in the FLSs of rats with CIA.

GRIA2, also known as glutamate ionotropic receptor AMPA type subunit 2 (GluA2), functions as a ligand-gated ion channel in the central nervous system and is crucial in excitatory synaptic transmission [33]. Glutamate is a non-essential amino acid that serves as a major bioenergetic substrate for both normal and neoplastic proliferating cells. Glutamatergic signaling activates a family of receptors, including metabotropic glutamate (mGluRs) and ionotropic glutamate receptors (iGluRs) [34]. Both receptor types are implicated in various neurological and psychiatric disorders and are promising therapeutic targets [35]. The activation of iGluRs in human synoviocytes has been shown to promote joint destruction by enhancing IL-6 expression [36]. Based on these findings, we hypothesize that the significant anti-inflammatory effects of baicalin in RA may be attributed to its ability to downregulate GluA2 expression.

γ-Aminobutyric acid (GABA) is a non-proteinogenic amino acid synthesized from glutamate by the enzyme glutamic acid decarboxylase [37]. It is found extensively in nature and is recognized as a potent bioactive compound with diverse physiological functions, such as modulating neuronal excitability and offering anti-RA effects [38,39]. The interplay between defective DNA repair, metabolic dysregulation, autoimmunity, and tissue inflammation in RA supports the exploration of metabolic modulation as a novel therapeutic strategy [40], and synovial membranes contain endogenous GABA, which participates in modulating the inflammatory response in RA [41]. GABA exerts its biological effects through three receptor types: ionotropic GABAA and GABAC receptors and the G protein-coupled GABAB receptor [42]. GABAA receptors, which are ligand-gated chloride channels, inhibit inflammatory progression in RA, while GABAB receptors act as susceptibility genes, modulating the immune response through the regulation of immune cells [43]. Dysregulating the expression and localization of α5-GABAA receptors has been associated with long-term impairments in learning and memory [44]. In this study, GABA is one of the three metabolites (MDs) regulated by baicalin, suggesting that the flavonoid may exert anti-inflammatory effects in part by enhancing GABA expression, thereby contributing to the regulation of inflammatory responses in RA.

Phosphatidylcholine (PC) and phosphatidylethanolamine (PE) are the most abundant phospholipids in mammalian cell membranes. Variations in the PC/PE molar ratio across tissues can influence energy metabolism and have been linked to disease progression [45,46], and, in mitochondria, they can affect energy production [47]. RA has been associated with elevated PE and reduced PC levels; thus, restoring the balance of these phospholipids may offer a means to alleviate RA-related inflammation [48]. In this study, baicalin was found to upregulate PC expression and downregulate PE expression in FLS cells, suggesting that its anti-inflammatory effects may be mediated through the normalization of the PC/PE ratio.

In summary, our data revealed that the RA-risk-associated retrograde endocannabinoid signaling pathway is the only one involved in both signaling and metabolic processes modulated by baicalin. The anti-inflammatory effects of the flavonoid in FLS cells are potentially mediated through an increase in GABA and PC levels; a decrease in the expression of GRIA2, GABRA5 and PE; and the maintenance of mitochondrial homeostasis. The specific molecular mechanism warrants more in-depth research in the future.

## 5. Conclusions

Our findings suggest that the baicalin-mediated alleviation of joint synovitis is closely related to the upregulation of GABA and PC; downregulation of GRIA2, GABRA5, and PE; and preservation of mitochondrial homeostasis within the retrograde endocannabinoid signaling pathway in FLSs. These results may help expand the range of clinical therapeutic strategies for RA.

## Figures and Tables

**Table 1:** The DEPs regulated by baicalin in Retrograde endocannabinoid signaling.

Protein Accession	Protein Description	Gene Name	Baicalin/Control *p*-Value	Regulated Type
P49821	NADH dehydrogenase [ubiquinone] flavoprotein 1	*Ndufv1*	2.88137 × 10^5^	Down
O75251	NADH dehydrogenase [ubiquinone] iron-sulfur protein 7	*Ndufs7*	5.63414 × 10^5^	Down
O95299	NADH dehydrogenase [ubiquinone] 1 alpha subcomplex subunit 10	*Ndufa10*	6.22403 × 10^5^	Down
Q9UI09	NADH dehydrogenase [ubiquinone] 1 alpha subcomplex subunit 12	*Ndufa12*	6.92543 × 10^5^	Down
P51970	NADH dehydrogenase [ubiquinone] 1 alpha subcomplex subunit 8	*Ndufa8*	8.36779 × 10^5^	Down
P28331	NADH-ubiquinone oxidoreductase 75 kDa subunit	*Ndufs1*	0.000191797	Down
O75380	NADH dehydrogenase [ubiquinone] iron-sulfur protein 6, mitochondrial	*Ndufs6*	0.000240219	Down
P42262	Glutamate receptor 2	*Gria2*	0.00032844	Down
O43677	NADH dehydrogenase [ubiquinone] 1 subunit C1	*Ndufc1*	0.000329508	Down
O95298	NADH dehydrogenase [ubiquinone] 1 subunit C2	*Ndufc2*	0.000434447	Down
O43181	NADH dehydrogenase [ubiquinone] iron-sulfur protein 4	*Ndufs4*	0.000528192	Down
P31644	Gamma-aminobutyric acid receptor subunit alpha-5	*Gabra5*	0.00061721	Down
O43920	NADH dehydrogenase [ubiquinone] iron-sulfur protein 5	*Ndufs5*	0.000840744	Down
O95167	NADH dehydrogenase [ubiquinone] 1 alpha subcomplex subunit 3	*Ndufa3*	0.00126853	Down
Q16718	NADH dehydrogenase [ubiquinone] 1 alpha subcomplex subunit 5	*Ndufa5*	0.001409079	Down
O95182	NADH dehydrogenase [ubiquinone] 1 alpha subcomplex subunit 7	*Ndufa 7*	0.001616009	Down
Q16795	NADH dehydrogenase [ubiquinone] 1 alpha subcomplex subunit 9	*Ndufa 9*	0.001807774	Down
P03897	NADH-ubiquinone oxidoreductase chain 3	*Mt-nd3*	0.004327636	Down
P03891	NADH-ubiquinone oxidoreductase chain 2	*Mt-nd2*	0.010683937	Down

**Table 2:** The DMs regulated by baicalin in Retrograde endocannabinoid signaling.

KEGG Compound ID	Name	Regulated Type
C00334	γ-aminobutyric acid	Up
C00157	Phosphatidylcholine	Up
C00350	Phosphatidylethanolamine	Down

## Data Availability

The mass spectrometry-based proteomics data presented in this study are available in the iProX partner repository at ProteomeXchange Consortium (http://proteomecentral.proteomexchange.org (accessed on 28 August 2025)), with the dataset identifier PXD045942. The mass spectrometry metabolomics data presented in this study are available from the China National Center at Bioinformatics (https://ngdc.cncb.ac.cn/omix/preview/kn6NqE4g (accessed on 28 August 2025)), under the dataset identifier OMIX009887.
